# Multifractality in Quasienergy Space of Coherent States as a Signature of Quantum Chaos

**DOI:** 10.3390/e23101347

**Published:** 2021-10-15

**Authors:** Qian Wang, Marko Robnik

**Affiliations:** 1 CAMTP-Center for Applied Mathematics and Theoretical Physics, University of Maribor, SI-2000 Maribor, Slovenia; Robnik@uni-mb.si; 2 Department of Physics, Zhejiang Normal University, Jinhua 321004, China

**Keywords:** quantum chaos, multifractal analysis, kicked top, coherent states

## Abstract

We present the multifractal analysis of coherent states in kicked top model by expanding them in the basis of Floquet operator eigenstates. We demonstrate the manifestation of phase space structures in the multifractal properties of coherent states. In the classical limit, the classical dynamical map can be constructed, allowing us to explore the corresponding phase space portraits and to calculate the Lyapunov exponent. By tuning the kicking strength, the system undergoes a transition from regularity to chaos. We show that the variation of multifractal dimensions of coherent states with kicking strength is able to capture the structural changes of the phase space. The onset of chaos is clearly identified by the phase-space-averaged multifractal dimensions, which are well described by random matrix theory in a strongly chaotic regime. We further investigate the probability distribution of expansion coefficients, and show that the deviation between the numerical results and the prediction of random matrix theory behaves as a reliable detector of quantum chaos.

## 1. Introduction

Quantum chaos plays a crucial role in many fields of physics, such as quantum statistics [1,2,3,4,5], quantum information science [6,7,8,9,10,11,12,13], and high-energy physics [14,15,16]. In particular, chaos of interacting quantum systems, dubbed as many-body quantum chaos, has attracted significant attention in recent years [17,18,19,20,21,22,23]. However, in contrast to classical chaos, which is well defined as the hypersensitivity to the initial condition [24,25,26], the definition of the quantum chaos in a time-dependent domain is still lacking, due to the fact that there is no quantum analog of classical trajectories in general quantum theory. In this regard, studies of OTOC (out-of-time ordered correlator) are highly relevant (see Section 3). Therefore, the questions of how the chaotic dynamics manifests itself in quantum systems and how to diagnose the quantum chaos immediately and naturally arise.

There are several ways to detect quantum chaos, which probe the effects of chaos on quantum systems from different aspects, the most popular one being the level spacing statistics [27,28,29,30,31,32,33,34]. The BGS conjecture [28] allows us to identify a given quantum system as chaotic system when its level spacing statistics is identical to the prediction of random matrix theory (RMT) [35]. Besides the level spacing statistics, the statistics of eigenvectors of quantum Hamiltonian can also be used as a benchmark to verify quantum chaos [32,36,37,38,39,40,41]. For quantum chaotic systems, their eigenfunction statistics is also well described by RMT.

A drawback of the above-mentioned quantum chaos indicators is that they only reveal the overall behaviors and cannot probe local properties of quantum chaotic systems. Since a generic system usually has a structured phase space with coexistence of regular and chaotic regions rather than a featureless fully developed chaotic region, it is highly desirable to investigate such quantities that enable us to analyze the local chaotic behaviors of a quantum system. With the help of coherent states (or localized wave packets), the local chaotic behaviors of quantum systems have been extensively explored in a variety of works [42,43,44,45,46,47,48,49]. Here, by considering the kicked top model, we are interested in how to reveal the phase space structures and the degree of chaos by means of multifractality of coherent states.

As a general phenomenon in nature, multifractality characterizes a wide range of complex phenomena from turbulence [50] to the chemistry [51] and financial markets [52]. It has been proven that the multifractal analysis also acts as a powerful tool to understand disorder induced metal-insulator transition in both single- and many-particle Hamiltonians [53,54,55,56,57,58]. The multifractality is also presented in the ground state of quantum many-body systems and determines the physics of ground state quantum phase transition [59,60,61,62]. In addition, multifractal analysis of quantum states of random matrix models [63,64,65,66,67], chaotic quantum many-body systems [68,69], and open quantum systems [70] have been studied. In the present work, the fractal properties of the coherent states are examined in order to identify both the local and global signatures of quantum chaos.

We perform multifractal analysis of coherent states by expanding them on the basis of the eigenstates of the Floquet operator. To quantify the character of multifractality, we consider the so-called multifractal dimensions $D_{q}$, which characterize the structure of a quantum state in Hilbert space. For fully chaotic states, $D_{q}=1$; for localized states, $D_{q}=0$ with q≥0; and for the multifractal states, $0<D_{q}<1$ is a function of *q* [58,69]. In the kicked top model, we show that the multifractal properties of coherent states strongly depend on the chaotic behavior of its classical counterpart. We find that the multifractal dimensions exhibit a similar transition as observed in phase-space portraits and Lyapunov exponents when the system varies from regular to mixed-phase and globally chaotic dynamics. In particular, we demonstrate that the structure of classical mixed phase space can be clearly distinguished by the properties of multifractal dimensions. We also show that coherent states within the strong chaotic regime become ergodic as the system size goes to infinity, as expected from RMT predictions. On the contrary, coherent states in a regular regime still behave as multifractal states even in the thermodynamic limit. By exploring the probability distribution of the expansion coefficients, we demonstrate why the multifratal dimensions of coherent states are not zero in the regular regime and why RMT predictions on the behavior of multifractal dimensions are reliable in the fully chaotic regime.

The remainder of this article is organized as follows. In Section 2, we introduce the kicked top model, derive the stroboscopic evolution of the angular momentum for both quantum and classical cases, and analyze the classical and quantum chaotic behaviors. In Section 3, we present our numerical results in detail for the multifractal analysis of coherent states and discuss the manifestation of phase space features and onset of chaos in behavior of multifractal dimensions. Finally, we make some concluding remarks and summarize our results in Section 4.

## 2. Kicked-Top Model

As a paradigmatic model for both theoretical [7,8,9,10,11,71,72,73,74,75,76,77,78] and experimental [79,80,81,82] studies of quantum chaos, the kicked top model consists of a larger spin with total angular momentum *j* whose dynamics is captured by the following Hamiltonian (throughout this work, ℏ=1) [10,71]:

(1) $$H=\alpha J_{x}+\frac{\kappa }{2j}J_{z}^{2}\sum_{n=-\infty }^{n=+\infty }\delta \left(t-n\right),$$

where $J_{a}\left(a=x,y,z\right)$ are the components of the angular momentum operator J. The first term in the Hamiltonian represents the free precession of the spin around the *x* axis at a rate α, while the periodic δ kicks with strength κ, the second term in Equation (1), periodically generates an impulsive rotation about the *z* axis by an angle $\left(\kappa /2j\right)J_{z}^{2}$, with *n* being the number of kicks. Here, the time period between two successive kicks has been set to unity. The time evolution operator corresponding to above Hamiltonian is the Floquet operator [71]

(2) $$F=\exp\left(-i\frac{\kappa }{2j}J_{z}^{2}\right)\exp\left(-i\alpha J_{x}\right).$$

In the numerical calculation, the Floquet operator should be expressed in a certain representation. To this end, we employ the Dicke states {|j,m〉;(m=−j,−j+1,…,j)}, that satisfy $J^{2}|j,m\rangle =j\left(j+1\right)|j,m\rangle$ and $j_{z}|j,m\rangle =m|j,m\rangle$. Then, the matrix elements of the Floquet operator are given by

(3) $$\langle j,m|F|j,m^{\prime }\rangle \;=\exp\left[-i\frac{\kappa }{2j}m^{2}\right]d_{mm^{\prime }}^{\left(j\right)}\left(\alpha \right),$$

where

(4) $$d_{mm^{\prime }}^{\left(j\right)}=\langle j,m|e^{-i\alpha J_{x}}|j,m^{\prime }\rangle =\sum_{k_{x}=-j}^{k_{x}=j}e^{-i\alpha k_{x}}\langle j,m|j,k_{x}\rangle \langle j,k_{x}|j,m^{\prime }\rangle ,$$

is the so-called Winger *d*-function [72], with $|j,k_{x}\rangle$ being the eigenstates of $J_{x}$, so that $J_{x}|j,k_{x}\rangle \;=k_{x}|j,k_{x}\rangle$ and $-j\leq k_{x}\leq j$. As the magnitude of spin operator is a conserved quantity, the matrix dimension is equal to 2j+1. Moreover, as the Floquet operator in Equation (2) also conserves parity $\Pi =e^{i\pi (J_{x}+j)}$, its matrix space can be further split into even- and odd-parity subspaces with dimensions $D_{even}=j+1$ and $D_{odd}=j$, respectively.

For an arbitrary initial state $|\psi_{0}\rangle$, the evolved state after the *n*th kick is given by

(5) $$|\psi_{n}\rangle \;=F^{n}|\psi_{0}\rangle .$$

The expectated values of the angular momentum operators are, therefore, evolved as follows

(6) $$\langle J_{a}\left(n\right)\rangle =\;\langle \psi_{n}|J_{a}|\psi_{n}\rangle \;=\;\langle \psi_{0}|F^{†,n}J_{a}\left(0\right)F^{n}|\psi_{0}\rangle ,$$

where $J_{a}\left(n\right)\left(a=x,y,z\right)$ denotes the *a*th components of the spin operator J at t=n. Hence, the stroboscopic evolution of the spin operators can be written as

(7) $$J_{a}\left(n+1\right)=F^{†}J_{a}\left(n\right)F.$$

By using the operator identity,

(8) $$e^{\lambda A}Be^{-\lambda A}=B+\lambda \left[A,B\right]+\frac{\lambda^{2}}{2}\left[A,\left[A,B\right]\right]+\ldots$$

the explicit form of the quantum iterated map reads [71,72,78]

$$\begin{array}{cc} J_{x}\left(n+1\right)= & \frac{1}{2}\left\{J_{x}\left(n\right)+i\left[J_{y}\left(n\right)\cos\alpha -J_{z}\left(n\right)\sin\alpha \right)\right\} \end{array}$$

(9) $$\begin{array}{cc} \; & \times \exp\left[i\frac{\kappa }{2j}\left\{2[J_{y}\left(n\right)\sin\alpha +J_{z}\left(n\right)\cos\alpha ]+1\right\}\right]\;+\;H.c.\\ J_{y}\left(n+1\right)= & \frac{1}{2i}\left\{J_{x}\left(n\right)+i\left[J_{y}\left(n\right)\cos\alpha -J_{z}\left(n\right)\sin\alpha \right)\right\} \end{array}$$

(10) $$\begin{array}{cc} \; & \times \exp\left[i\frac{\kappa }{2j}\left\{2[J_{y}\left(n\right)\sin\alpha +J_{z}\left(n\right)\cos\alpha ]+1\right\}\right]\;+\;H.c. \end{array}$$

(11) $$\begin{array}{cc} J_{z}\left(n+1\right)= & J_{y}\left(n\right)\sin\alpha +J_{z}\cos\alpha . \end{array}$$

### 2.1. Classical Kicked Top

The classical counterpart of the kicked top model can be obtained in the limit j→∞. To show this, we first introduce the scaled spin operators $S_{a}=J_{a}/j$, which behave as classical variables due to the vanishing commutators between them as j→∞. Then, by factorizing the mean values of the products of the angular momentum operators as $\langle J_{a}J_{b}\rangle /j^{2}=S_{a}S_{b}$ [9,78,83], it is straightforward to find that the stroboscopic map of the classical angular momentum can be written as [10]

(12) $$\left[\begin{array}{c} S_{x}\left(n+1\right)\\ S_{y}\left(n+1\right)\\ S_{z}\left(n+1\right) \end{array}\right]\;=M\left[\begin{array}{c} S_{x}\left(n\right)\\ S_{y}\left(n\right)\\ S_{z}\left(n\right) \end{array}\right]\;=\;\left(\begin{array}{ccc} \cos\Xi_{n} & -\cos\alpha \sin\Xi_{n} & \sin\alpha \sin\Xi_{n}\\ \sin\Xi_{n} & \cos\alpha \cos\Xi_{n} & -\sin\alpha \cos\Xi_{n}\\ 0 & \sin\alpha & \cos\alpha \end{array}\right)\left[\begin{array}{c} S_{x}\left(n\right)\\ S_{y}\left(n\right)\\ S_{z}\left(n\right) \end{array}\right],$$

where $\Xi_{n}=\kappa \left[S_{y}\left(n\right)\sin\alpha +S_{z}\left(n\right)\cos\alpha \right]$. As the classical angular momentum $S=(S_{x},S_{y},S_{z})$ is a unit vector, it can be parametrized in terms of the azimuthal angle θ and polar angle ϕ as S=(cosϕsinθ,sinϕsinθ,cosθ). Hence, the classical phase space is a two dimensional space with variables $\phi =\arctan(S_{y}/S_{x})$ and $\theta =\arccos(S_{z})$.

**Figure 1:**
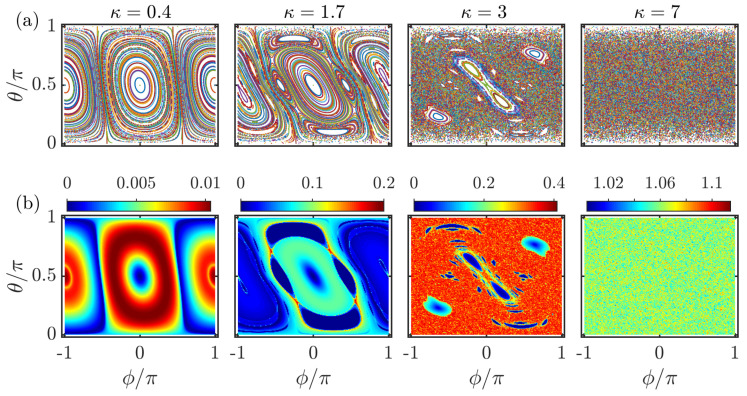
Row (**a**): Phase-space portraits of the classical kicked top. The classical variables (ϕ,θ) are plotted for 289 random initial conditions, each evolved for 300 kicks. Row (**b**): Color scaled plots of the largest Lyapunov exponent of the classical kicked top for different initial conditions. The largest Lyapunov exponents are calculated on a grid with 200×200 initial conditions, each evolved for 5000 kicks. The different columns correspond to (from left to right): κ=0.4,1.7,3 and κ=7. Other parameter: α=4π/7. All quantities are dimensionless.

It is known that the classical kicked top model is integrable at κ=0 and shows increasingly chaotic behavior with increasing κ. To visualize how the value of κ affects the dynamics of the classical kicked top model, the phase-space portraits for different κ values with α=4π/7 are plotted in Figure 1a. The phase space is largely dominated by the regular orbits at small values of κ, as shown in the first two columns of Figure 1a. The phase space becomes mixed with regular regions coexisting with the chaotic sea as κ is increased; see the third column of Figure 1a. For κ increasing further, the phase space is fully covered by chaotic trajectories, and there is no visible regular island in the last column of Figure 1a.

To quantify the chaotic features observed in Figure 1a, we investigate the behavior of the largest Lyapunov exponent of the classical map in Equation (12). The largest Lyapunov exponent measures the rate of divergence between two infinitesimally close orbits of a dynamical system [78,84,85]. The largest Lyapunov exponent, therefore, estimates the level of chaos. For the classical map in Equation (12), the largest Lyapunov exponent is defined as [86]

(13) $$\lambda_{+}=\lim_{n\to \infty }\frac{1}{n}\ln\left[\frac{\left||\delta S(n)|\right|}{\left||\delta S(0)|\right|}\right],$$

where the Oseledets ergodic theorem [87] guarantees the existence of the limit. Here, the 3-dimensional vector δS(n) is the tangent vector associated with S(n) and satisfies the following tangent map

(14) $$\delta S\left(n+1\right)=T\left[S\left(n\right)\right]\delta S\left(n\right)=\;\left[\frac{\partial S(n+1)}{\partial S(n)}\right]\delta S\left(n\right),$$

with initial condition δS(0). Then, the largest Lyapunov exponent of the classical kicked top can be calculated as [78,88]

(15) $$\lambda_{+}=\ln\left[\lim_{n\to \infty }{\left(\mu_{+}\right)}^{1/n}\right],$$

where $\mu_{+}$ denotes the largest eigenvalue of the matrix $\prod_{\ell =1}^{n}T\left[S\left(\ell \right)\right]$. In the limit of strong chaotic dynamics κ→∞, it has been found that the largest Lyapunov exponent has the following approximate expression [88]

(16) $$\lambda_{+}^{\infty }=\ln\left(\kappa \sin\alpha \right)-1,$$

where sinα>0. It has been shown that the classical map in Equation (12) has no fully developed chaos for the cases of α=kπ with k=0,1,2,… [89]. This is due to the fact that the angle θ either keeps fixed at $\arccos[S_{z}\left(0\right)]$ or oscillates between $\arccos[S_{z}\left(0\right)]$ and $\pi -\arccos[S_{z}\left(0\right)]$ in these cases. On the other hand, the cases of α=(2k+1)π/2 allow the strongest chaotic dynamics for classical kicked top.

In the row (b) of Figure 1, the largest Lyapunov exponents for different initial points in the ϕ−θ plane corresponding to the same values of κ used in row (a) are plotted. By comparing Figure 1a,b, we found that the largest Lyapunov exponents demonstrated remarkable resemblance with the corresponding classical phase portraits. The dominated regular orbits at small κ in the phase space leads to the tiny values of the largest Lyapunov exponents, as seen in the first two columns of Figure 1b. However, the fully chaotic phase space at κ=7 is clearly manifested by larger values of the largest Lyapunov exponent, which shows a uniform distribution in the phase space (see the last column in Figure 1b). In particular, the regular regions in the mixed phase space are identified by $\lambda_{+}=0$, as depicted in the third column of Figure 1b.

To further reveal the effect of the kicking strength on the overall degree of chaos in the classical kicked top, we consider the phase-space-averaged largest Lyapunov exponent ${\bar{\lambda }}_{+}$, which is defined as

(17) $${\bar{\lambda }}_{+}=\frac{1}{4\pi }\int dS\lambda_{+},$$

where dS=sinθdθdϕ is the area element (or Haar measure) in the phase space [90]. It is interesting to note that ${\bar{\lambda }}_{+}$ can be considered as the rescaled Kolmogorov–Sinai (KS) entropy $h_{KS}$ [91,92], as according to the Pesin formula [93], $h_{KS}$ of the kicked top model is equal to the sum of the largest Lyapunov exponents, so that $h_{KS}=\int dS\lambda_{+}$.

**Figure 2:**
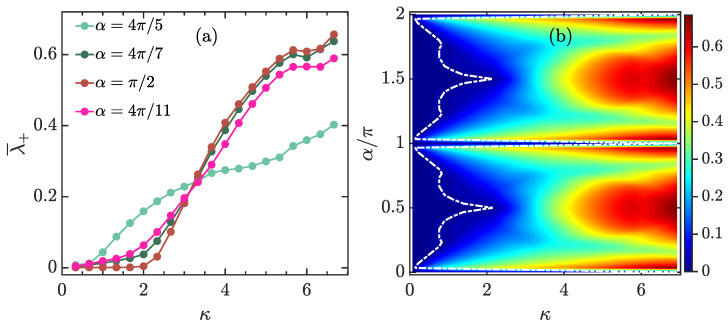
(**a**): Phase-space-averaged largest Lyapunov exponent ${\bar{\lambda }}_{+}$ as a function of κ for several values of α. (**b**): ${\bar{\lambda }}_{+}$ as a function of κ and α. The averaged largest Lyapunov exponents are calculated by averaging $\lambda_{+}$ over 40,000 different initial conditions, each evolved for 5000 kicks. In (**b**), the white dot-dashed curve corresponds to the values of $\kappa_{c}$ at which ${\bar{\lambda }}_{+}=0.002$. All quantities are dimensionless.

We plot ${\bar{\lambda }}_{+}$ as a function of κ for different values of α in Figure 2a. From this figure, we see that ${\bar{\lambda }}_{+}$ exhibits a rapid growth with increasing κ when $\kappa >\kappa_{c}$, regardless of the value of α. Here, $\kappa_{c}$ is defined as a threshold at which ${\bar{\lambda }}_{+}{|}_{\kappa =\kappa_{c}}=0.002$. This implies the onset of chaos in the classical kicked top for $\kappa >\kappa_{c}$. We further observe that with change of α there is a variation in the value of $\kappa_{c}$. Figure 2b depicts ${\bar{\lambda }}_{+}$ as a function of α and κ. We make several observations from Figure 2b. First, the behavior of ${\bar{\lambda }}_{+}$ shows a symmetry with respect to α=π. This is because for the classical map in Equation (12), α→α+π is equivalent to the transformation $S_{x}\to -S_{x}$, $S_{y}\to -S_{y}$ and $S_{z}\to -S_{z}$, which keeps the largest Lyapunov exponent unchanged [90]. Second, as the classical kicked top is integrable at α=0,π,2π, we have ${\bar{\lambda }}_{+}=0$ for these values of α, regardless of κ. Finally, for 0<α<π, even though the sharp growth behavior of ${\bar{\lambda }}_{+}$ with increasing κ for $\kappa >\kappa_{c}$ is independent of α, there is a strong dependence of $\kappa_{c}$ on α, as we have already seen in Figure 2a. The white dot-dashed curve in Figure 2b shows how α affects the value of $\kappa_{c}$. By confining to the range 0<α<π, we see that $\kappa_{c}$ is firstly increased with increasing α and it reaches its maximal value at α=π/2, and then starts to decrease as α increases further. The maximal value of $\kappa_{c}$ at α=π/2 results from the additional symmetry of the system [71], which leads to the onset of chaos occurring later than in the cases with other values of α. Without loss of general qualitative behavior, in the remainder of this work, we fixed α=4π/7.

### 2.2. Quantum Chaos of the Kicked-Top Model

The classical chaotic features discussed above are associated with quantum chaotic behavior in quantum kicked top model. The quantum character of chaos can be detected in several ways, such as the statistical properties of eigenvalues and eigenvectors [30,31,32], the dynamical features of entanglement entropy [8,11,94,95,96,97], the decay in fidelity [98], the correlation hole in survival probability [99], and, in particular, the dynamics of the out-of-time-ordered correlator (OTOC) [11,96,100,101,102,103]. Among them, one of the most widely used is energy-level statistics of the quantum Hamiltonian. It is known that integrable systems allow level crossings, which give rise to Poisson distribution of the nearest level spacings [27]. On the other hand, based on the work of Wigner [104], Bohigas, Giannoni, and Schmit conjecture predicts that the energy levels in chaotic systems should exhibit level repulsion and that the distribution of the nearest level spacings follows the Wigner–Dyson distribution [28]. Here, we would like to point out that the explanation of the BGS conjecture has been first investigated through a two-point spectral correlation function [31,105], and then extended to *n*-point correlations with n>2 [106,107,108].

The spectral statistics for a periodically driven quantum system can be analyzed through the quasienergies (or eigenphases) of the Floquet operator [109]. The quasienergy spectrum of the kicked top model is obtained from the eigenphases of the Floquet operator F in Equation (2), and are defined as

(18) $$F|\nu_{i}\rangle \;=e^{i\nu_{i}}|\nu_{i}\rangle ,$$

where $\nu_{i}$ denotes the *i*th eigenphase of F with corresponding eigenstate $|\nu_{i}\rangle$. As $\left\{\nu_{i}\right\}$ are 2π periodic, we restrict them within the principal range [−π,π).

**Figure 3:**
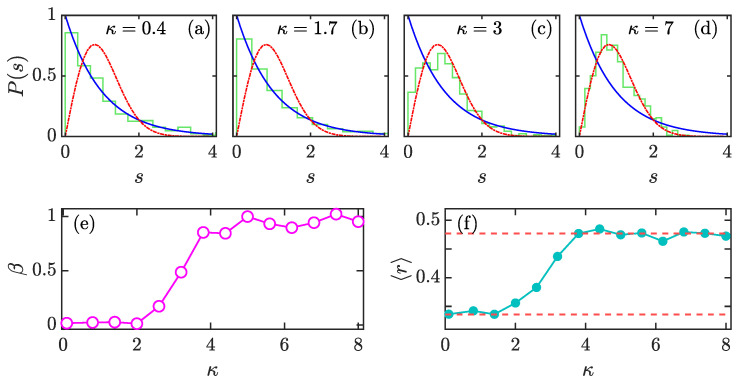
Level spacing distributions of the kicked top model for (**a**) κ=0.4, (**b**) κ=1.7, (**c**) κ=3, and (**d**) κ=7. The Poisson distribution is plotted as a blue solid curve, and the red dot-dashed curve denotes the Wigner–Dyson statistics. (**e**) The level repulsion exponent β as a function of κ. (**f**) Averaged level spacing ratio 〈r〉 as a function of κ. The upper (bottom) red dashed line indicates ${\langle r\rangle }_{COE}\approx 0.527$ (${\langle r\rangle }_{P}\approx 0.386$). Other parameters: j=1000 and α=4π/7. All quantities are dimensionless.

Numerically, the spectral analysis is performed as follows. Firstly, we diagonalize F on the basis of ${\{|j,m\rangle \}}_{m=-j}^{m=j}$ and only consider the quasienergies for the Floquet eigenstates with even parity. Then, by arranging $\left\{\nu_{i}\right\}$ in ascending order, we define the gap between two consecutive levels as $d_{i}=\nu_{i+1}-\nu_{i}$. Finally, we calculate the distribution P(s) of the normalized level spacings $s_{i}=d_{i}/\langle d\rangle$ [31], where 〈d〉 denotes the mean spacing. The dependence of P(s) on κ is shown in Figure 3a–d. Obviously, with increasing κ, the level spacing distribution P(s) undergoes a transition from Poisson statistics $P_{P}\left(s\right)=e^{-s}$ to Wigner–Dyson statistics $P_{WD}\left(s\right)=\left(\pi /2\right)s\exp\left(-\pi s^{2}/4\right)$. This is consistent with the classical dynamics observed in Figure 1.

To estimate the degree of chaos in Floquet spectrum of the kicked top model, we fit P(s) to the so-called Brody distribution defined as [30]

(19) $$P_{B}\left(s\right)=b_{\beta }\left(\beta +1\right)s^{\beta }\exp\left[-b_{\beta }s^{\beta +1}\right],$$

where the factor $b_{\beta }$ can be calculated as

(20) $$b_{\beta }={\left[\Gamma \left(\frac{\beta +2}{\beta +1}\right)\right]}^{\beta +1},$$

where Γ(x) is the gamma function. The parameter β, which measures the degree of repulsion between levels, is the level repulsion exponent and varies in the range 0≤β≤1. For β=0, the Brody distribution reduces to Poisson distribution, while it becomes Wigner–Dyson distribution at β=1. Therefore, the larger β is, the stronger the chaotic spectrum is. Figure 3e plots β as a function of κ with j=1000 and α=4π/7. The behavior of β nicely agrees with spectral analysis: for κ≲2, we have β≈0, implying the Poisson distribution of P(s), while β approaches unity when κ≳5, suggesting that the quasienergy levels have the strongest repulsion and that P(s) is the Wigner–Dyson distribution. It is worth pointing out that the transition region defined as 0<β<1 corresponds to the classical mixed-phase space with regular regions embedded in the chaotic sea. (see, e.g., the third column in Figure 1). More details about the spectral statistics in the transition region between integrability and chaos can be found in [110] and references therein. We only mention that here the Berry–Robnik level spacing distribution [111] is not yet manifested, as we are not yet in sufficiently deep semiclassical regime and observe Brody distribution instead.

Besides the level spacing distribution, the mean ratio of consecutive level spacing is another widely used detector of quantum chaos. Given the level spacing $\left\{d_{i}\right\}$, the mean ratio of level spacing is defined as [33,34]

(21) $$\langle r\rangle =\frac{1}{N}\sum_{i=1}^{N}r_{i},\;r_{i}=\min\left(\frac{1}{\delta_{i}},\delta_{i}\right),$$

where N is the total number of $r_{i}$ and $\delta_{i}=d_{i+1}/d_{i}$. It has been demonstrated that the averaged ratio of level spacing, 〈r〉, acts as an indicator of spectral statistics. For regular systems with Poisson statistics ${\langle r\rangle }_{P}\approx 0.386$, while ${\langle r\rangle }_{COE}\approx 0.527$ for circular orthogonal ensemble (COE) of random matrices [34]. We plot 〈r〉 as a function of κ for α=4π/7 in Figure 3f. One can see that 〈r〉 exhibits a crossover from ${\langle r\rangle }_{P}$ to ${\langle r\rangle }_{COE}$ with κ increasing. This is in agreement with the behavior of P(s), as observed in Figure 3a–d. Moreover, we notice that the behavior of 〈r〉 is similar to the level of the repulsion exponent β (cf. Figure 3e).

Even though the level statistics becomes a standard probe in the studies of quantum chaos, it cannot detect the local chaotic features in quantum systems. In order to characterize the phase space structure and get more insight into the quantum-classical correspondence, we consider the multifractal properties of the coherent states in the following.

### 2.3. Coherent States

The coherent states have wide applications in many fields [112,113,114,115,116]. As the uncertainty of coherent states tends to zero in the classical limit, one can expect that the phase space structure and the quantum-classical correspondence can be unveiled through appropriate properties of coherent states. For our purpose, we use the generalized SU(2) coherent spin states, which are constructed by applying an appropriate rotation on the state |j,j〉 [113,114],

(22) $$|\vartheta ,\varphi \rangle =\exp\;\left[i\vartheta (J_{x}\sin\varphi -J_{y}\cos\varphi )\right]|j,j\rangle ,$$

where ϑ,φ provide the orientation of J. Further simplification of |ϑ,φ〉 is available by performing Taylor expansion and the final result is given by [114,117]

(23) $$|\vartheta ,\varphi \rangle =\frac{e^{\zeta J_{-}}}{{\left(1+|\zeta \right|}^{2}{)}^{j}}|j,j\rangle =\sum_{m=-j}^{j}\frac{\zeta^{j-m}}{{\left(1+|\zeta \right|}^{2}{)}^{j}}\sqrt{\frac{(2j)!}{(j+m)!(j-m)!}}|j,m\rangle ,$$

where $J_{-}=J_{x}-iJ_{y}$ and $\zeta =\tan\left(\vartheta /2\right)e^{i\varphi }$. It is straightforward to show that the uncertainty of the coherent spin state |ϑ,φ〉 in Equation (23) vanishes as j→∞.

Here, it is worth noting that the coherent states have been exploited to explore the quantum and classical structures of the kicked-top model in several works [90,118]. The quantum-classical correspondence for various structures has been established. In particular, those works have shown that some valuable information of the scarred eigenstates, which are localized along the classical unstable periodic orbits, can be extracted from the properties of the coherent states.

## 3. Multifractality of Coherent States

The notion of multifractality was originally introduced to describe complex fluctuations observed in fluid turbulence [50]. It has been recognized as a valuable tool to analyze a variety of classical complex phenomena. Moreover, it has been found that the multifractal phenomenon was also visible in a quantum state. Quantum state multifractality reflects its unusual statistical properties and has attracted much attention as it plays a prominent role in the phase transitions of different quantum systems [55,56,57,58,59,60,61,69,119]. The characterization of the multifractality is quantified by the so-called generalized fractal dimensions, denoted by $D_{q}$. To define $D_{q}$, let us consider a quantum state |Φ〉 expanded in a given orthonormal basis {|k〉} with dimension N,

(24) $$|\Phi \rangle =\sum_{k=1}^{N}c_{k}|k\rangle ,$$

where $c_{k}=\langle k|\Phi \rangle$ and satisfies $\sum_{k}{\left|c_{k}\right|}^{2}=1$. Then, $D_{q}$ is defined as [53,69]

(25) $$D_{q}=\frac{S_{q}}{\ln N}\;\mathrm{and}\;S_{q}=\frac{1}{1-q}\ln\left(\sum_{k=1}^{N}{\left|c_{k}\right|}^{2q}\right),$$

where $S_{q}$ is the Rényi entropy (or participation entropy). For finite N, the values of $D_{q}$ are defined in the interval $D_{q}\in \left[0,1\right]$ and decrease with increasing *q* for q≥0 [68]. The fractal dimensions, $D_{q}^{\infty }$, are obtained as N→∞, so that $D_{q}^{\infty }=\lim_{N\to \infty }D_{q}$ [53,68]. The degree of ergodicity of a quantum state in Hilbert space is measured by the fractal dimensions. For a perfectly localized state $D_{q}^{\infty }=0$ for q>0, whereas $D_{q}^{\infty }=1\left(\forall q\right)$ corresponds to an ergodic state. The multifractal states are the extended non-ergodic states and identified by $0<D_{q}^{\infty }<1$.

Among all $D_{q}$, we focus on the cases where q=1,2, and *∞*. As the Rényi entropy reduces to the Shannon entropy, $S_{1}=-\sum_{k}|c_{k}{|}^{2}\ln{\left|c_{k}\right|}^{2}$, in the limit q→1, the dimension $D_{1}$, also known as information dimension, controls the scaling of Shannon information entropy. For q=2, $S_{2}=\ln(\sum_{k}|c_{k}{{|}^{4})}^{-1}$ is the logarithm of the well-known participation ratio [57,58,120], which measures the degree of delocalization of the state in Hilbert space. Hence, the exponent $D_{2}$ quantifies the scaling of the participation ratio. At q=∞, the Réyni entropy turns into $S_{\infty }=-\ln p_{m}$ with $p_{m}=\max_{k}{\left|c_{k}\right|}^{2}$ and $D_{\infty }=-\ln p_{m}/\ln N$, determining the extreme value statistics of the intensities of the quantum state.

In our study, we analyze the multifractal properties of the generalized SU(2) coherent spin states (cf. Equation (23)) in the eigenvectors of the Floquet operator. Therefore, we first expand |ϑ,φ〉 on the basis of $\left\{|\nu_{i}\rangle \right\}$ as follows

(26) $$|\vartheta ,\varphi \rangle =\sum_{i}w_{i}|\nu_{i}\rangle ,$$

where $w_{i}=\langle \nu_{i}|\vartheta ,\varphi \rangle$ is the overlap between the basis vector $|\nu_{i}\rangle$ and the coherent state |ϑ,φ〉, fulfilling the normalization condition $\sum_{i}{\left|w_{i}\right|}^{2}=1$. Then, using Equation (25), the fractal dimensions are calculated for coherent states that are centered at different points (θ,ϕ) of the classical phase space.

**Figure 4:**
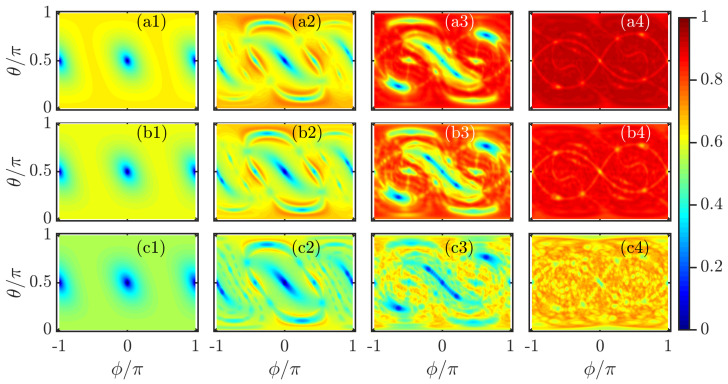
Color scaled plot of multifractal dimensions $D_{q}$ for (**a1**–**a4**) q=1, (**b1**–**b4**) q=2, and (**c1**–**c4**) q=∞, calculated on a grid of 100×100 coherent states. The different columns correspond to (from left to right): κ=0.4, κ=1.7, κ=3, and κ=7. Other parameters: j=150 and α=4π/7. All quantities are dimensionless.

In Figure 4, we plot $D_{1},D_{2}$, and $D_{\infty }$ as a function of ϕ and θ for different kicking strengths κ. By comparing with the classical phase space portraits in Figure 1a, we observe that the underlying classical dynamics has strong effects on the properties of the fractal dimensions. The regular regions around the fixed points give rise to $D_{q}\approx 0$, indicating the coherent states located at these points are the localized states, as seen in the first and second columns of Figure 4. In the chaotic phase space, the fractal dimensions have larger values and exhibit an approximately uniform distribution in the phase space (see the last column of Figure 4). These features imply that the coherent states have high degree of ergodicity for large kicking strength. For the mixed phase space, it is evident from the third column of Figure 4 that the regular regions are identified by smaller fractal dimensions, while larger $D_{q}$ correspond to the chaotic sea. The obvious correspondence between the fractal dimension and the classical phase space dynamics and the Lyapunov exponents, as shown in Figure 1a,b, suggests that $D_{q}$ are particularly useful to detect the signatures of quantum chaos. We further notice that $0<D_{q}<1$ still holds even if the system is governed by regular dynamics.

To further demonstrate that $D_{q}$ can enable us to discern the regular and chaotic characters of the quantum system, we assess the phase-space-averaged fractal dimensions, defined as

(27) $${\bar{D}}_{q}=\frac{1}{4\pi }\int dSD_{q}.$$

**Figure 5:**
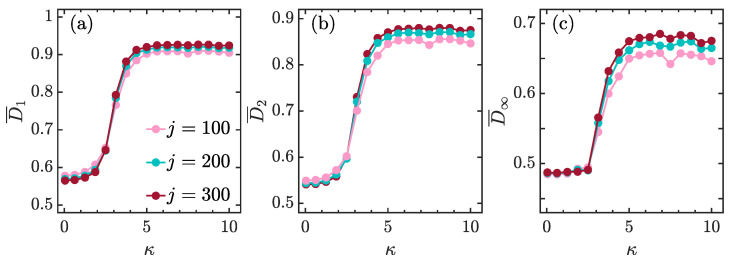
The variation in phase-space-averaged multifractal dimensions ${\bar{D}}_{1}$ (**a**), ${\bar{D}}_{2}$ (**b**), and ${\bar{D}}_{\infty }$ (**c**) with kicking strength κ for different *j* are denoted by color scales. The phase space average is performed over $10^{4}$ coherent states in phase space. Other parameters: α=4π/7. All quantities are dimensionless.

Figure 5a–c show, respectively, ${\bar{D}}_{1},{\bar{D}}_{2}$ and ${\bar{D}}_{1}$ as a function of κ for different system sizes *j*. We see that the dependence of fractal dimensions on κ is similar for different *j*. The fractal dimensions change slowly with increasing κ for smaller κ and exhibit a rapid growth as soon as κ>2. Then, ${\bar{D}}_{q}$ eventually approach their saturation values when κ>5. Moreover, we also observe that ${\bar{D}}_{q}$ are almost independent of *j* for κ<2, while they increase with increasing *j* as long as κ>5.

**Figure 6:**
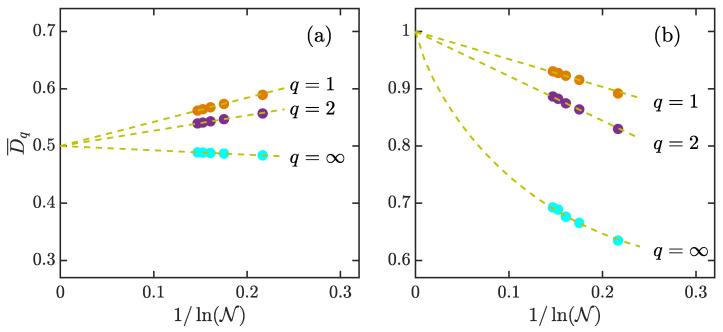
Phase-space-averaged fractal dimensions ${\bar{D}}_{q}$ with q=1,2,∞ versus 1/lnN for κ=0.4 (**a**) and κ=7 (**b**). Here, N denotes the dimension of Hilbert space. ${\bar{D}}_{q}$ were calculated from $10^{4}$ coherent states in phase space. Dashed lines in panel (**a**) are of the form $1/2-f_{q}/\ln N$, with $f_{1}=0.421,f_{2}\;=\;0.267$ and $f_{\infty }=-0.0758$. In panel (**b**), dashed lines for q=1,2 are of the form $1-g_{q}/\ln N$ with $g_{1}=0.484$ and $g_{2}=0.779$, while the dashed line for q=∞ is given by $1\;-\;g_{\infty }\ln\left(\ln N\right)/\ln N$ with $g_{\infty }=1.097$. Other parameters: α=4π/7. All quantities are dimensionless.

In Figure 6, we plot the scaling of ${\bar{D}}_{q}$ with 1/lnN for κ=0.4 and κ=7. Here, N is the Hilbert space dimension of the system. For the regular regime with κ=0.4 (Figure 6a), ${\bar{D}}_{q}$ follow the linear scaling of the form ${\bar{D}}_{q}=1/2-f_{q}/\ln N$ with $f_{q}$ depending on the value of *q*. In particular, the scaling behaviors of ${\bar{D}}_{q}$ imply that ${\bar{D}}_{q}$ tend to 1/2 rather than zero as N→∞. On the other hand, according to RMT, ${\bar{D}}_{q}$ in a fully chaotic regime obey the following asymptotic behavior [68,69,121]

(28) $${\bar{D}}_{q}^{s}=\{\begin{array}{cc} 1-\frac{g_{q}}{\ln N} & \mathrm{for}\;q=1,2,\\ 1-g_{\infty }\frac{\ln(\ln N)}{\ln N} & \mathrm{for}\;q=\infty . \end{array}$$

As the kicked top becomes a strongly chaotic system at larger κ, one can expect that the scaling behaviors of ${\bar{D}}_{q}$ should be in agreement with the above results and should approach unity in the thermodynamic limit. This is indeed what we see in panel (b) of Figure 6, which shows how ${\bar{D}}_{q}$ vary with 1/lnN at κ=7. A good agreement between the numerical data and ${\bar{D}}_{q}^{s}$ in Equation (28) leads us to conclude that the coherent states in strongly chaotic regimes become ergodic in the eigenstates of the Floquet operator.

More insights into the ergodic property of the coherent states in the eigenvectors of the Floquet operator can be obtained through the statistics of the rescaled expansion coefficients $\left\{x_{i}=N|w_{i}{|}^{2}\right\}$. For fully chaotic systems, it has been demonstrated that the probability distribution of $\left\{x_{i}\right\}$ for different ensembles are unified in the $\chi_{\nu }^{2}$ distribution, as shown in [35,39,40,41,42],

(29) $$P_{\nu }\left(x\right)={\left(\frac{\nu }{2\langle x\rangle }\right)}^{\nu /2}\frac{x^{\nu /2-1}}{\Gamma (\nu /2)}\exp\left(-\frac{\nu x}{2\langle x\rangle }\right),$$

where 〈x〉 is the mean value of $\left\{x_{i}\right\}$ and ν=1,2,4 for orthogonal, unitary, and symplectic ensembles, respectively. In particular, $P_{\nu }\left(x\right)$ turns into the well-known Porter–Thomas distribution [37] when ν=1. The width of the distribution becomes narrower with increasing ν, indicating the larger of the value of ν, the smaller the fluctuations of $\left\{x_{i}\right\}$.

For the coherent state considered here, the expansion coefficients are complex numbers, and their distribution in the fully chaotic regime should be expected to be given by $\chi_{\nu }^{2}$ distribution with ν=2 [38,122]. Moreover, due to the large amount of small coefficients, we explore the distribution of $\left\{\ln x_{i}\right\}$ instead of $\left\{x_{i}\right\}$. From Equation (29), $P_{\nu }\left(\ln x\right)$, is given by

(30) $$P_{\nu }\left(\ln x\right)=\frac{P_{\nu }\left(x\right)}{d\ln x/dx}={\left(\frac{\nu }{2\langle x\rangle }\right)}^{\nu /2}\frac{x^{\nu /2}}{\Gamma (\nu /2)}\exp\left(-\frac{\nu x}{2\langle x\rangle }\right).$$

The relation $P_{\nu }\left(\ln x\right)=xP_{\nu }\left(x\right)$ implies that P(lnx) has the maximal value at x=〈x〉.

**Figure 7:**
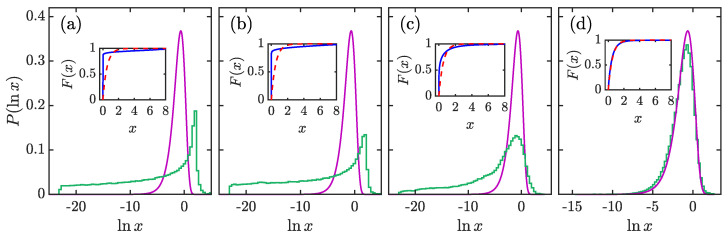
Histograms of P(lnx) for κ=0.4 (**a**), κ=1.7 (**b**), κ=3 (**c**), and κ=7 (**d**). The purple solid lines in the main panels denote $P_{2}\left(\ln x\right)$ [cf. Equation (30)]. The inset in each panel plots their cumulative distributions with blue solid curve corresponds to numerical result, while the red dashed curve represents $F_{2}\left(x\right)$ (cf. Equation (32)). P(lnx) has been computed from $10^{4}$ coherent states in phase space. Other parameters: j=150 and α=4π/7. All quantities are dimensionless.

In the main panels of Figure 7, we show P(lnx) and compare with $P_{2}\left(\ln x\right)$ for several values of κ. The numerical data are obtained from $10^{4}$ coherent states that are uniformly located in the phase space. As expected, for the regular case with smaller κ values, the larger number of small coefficients leads to larger fluctuations around its averaged value and a greater deviation from $P_{2}\left(\ln x\right)$, as shown in Figure 7a,b. However, the peak of P(lnx) around lnx∼2 results in moments of P(x) that are *q*-dependent, which means non-zero multifractal dimensions $D_{q}\left(q>0\right)$ at smaller κ values. With further increasing κ, the distribution of P(lnx) shifts its location to larger values of lnx and becomes narrower (Figure 7c). For even larger κ values, the distribution P(lnx) eventually converges to $P_{2}\left(\ln x\right)$, as visible in Figure 7d. Here, we would like to point out that the peaks observed in panels (a) and (b) of Figure 7 have nothing to do with the regularity and/or chaos. In fact, their appearance depends on the computation basis that we used to expand the quantum state, as has been stressed in [41]. The regularity of a system is only manifested in the long flat tail of P(lnx).

The convergence between the distributions of P(x) and $P_{2}\left(x\right)$ as κ increases is also confirmed in the behavior of the corresponding cumulative distributions. For the distribution P(x), the cumulative distribution is defined as

(31) $$F\left(x\right)=\int_{0}^{x}P\left(t\right)dt,$$

while the cumulative distribution of $P_{\nu }\left(x\right)$ is given by

(32) $$F_{\nu }\left(x\right)=\int_{0}^{x}P_{\nu }\left(t\right)dt=\frac{\gamma \left[\nu /2,\nu x/(2\langle x\rangle )\right]}{\Gamma (\nu /2)},$$

where $\gamma \left(s,x\right)=\int_{0}^{x}t^{s-1}e^{-t}dt$ is the lower incomplete gamma function. The insets in Figure 7 show F(x) and $F_{2}\left(x\right)$ for different κ values. It can be seen that the deviation between F(x) and $F_{2}\left(x\right)$ decreases with increasing κ, in accordance with the behavior of P(lnx) observed in the main panels.

To quantify the distance between P(x) and $P_{2}\left(x\right)$, we use two different deviation measures, namely the square root of the Kullback–Leibler divergence (SKLD) [123,124] and the root-mean-square error (RMSE) [125,126]. For the observed distribution P(x) and predicted distribution $P_{\nu }\left(x\right)$, the SKLD (RMSE), denoted as $D_{KL}^{\left(\nu \right)}\left(R_{d}^{\left(\nu \right)}\right)$, measures the difference between observed and predicted probability (cumulative) distributions. The definitions of SKLD and RMSE are, respectively, given by

(33) $$\begin{array}{cc} \; & D_{KL}^{\left(\nu \right)}={\left\{\int_{x_{0}}^{x_{m}}P\left(x\right)\ln\left[\frac{P(x)}{P_{\nu }\left(x\right)}\right]dx\right\}}^{1/2}, \end{array}$$

(34) $$\begin{array}{cc} \; & R_{d}^{\left(\nu \right)}={\left\{\frac{1}{x_{m}-x_{0}}\int_{x_{0}}^{x_{m}}\left[F\left(x\right)-F_{\nu }\left(x\right)\right]dx\right\}}^{1/2}, \end{array}$$

where $x_{0}$ and $x_{m}$ are the minimum and maximum values of $\left\{x_{i}\right\}$, respectively. Both $D_{KL}^{\left(\nu \right)}$ and $R_{d}^{\left(\nu \right)}$ are defined in the interval $D_{KL}^{\left(\nu \right)},\;R_{d}^{\left(\nu \right)}\in \left[0,\infty \right)$. When $D_{KL}^{\left(\nu \right)}=R_{d}^{\left(\nu \right)}=0$, we have $P\left(x\right)=P_{\nu }\left(x\right)$, whereas larger $D_{KL}^{\left(\nu \right)},\;R_{d}^{\left(\nu \right)}$ values imply a larger deviation between P(x) and $P_{\nu }\left(x\right)$.

**Figure 8:**
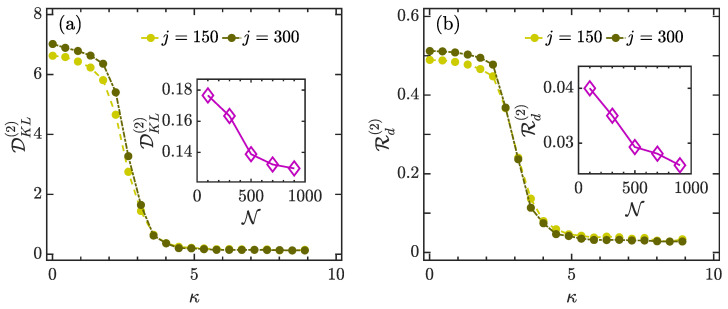
Panel (**a**): $D_{KL}^{\left(2\right)}$ as a function of κ for different system sizes. Inset: $D_{KL}^{\left(2\right)}$ as a function of Hilbert space dimension N with κ=8. Panel (**b**): $R_{d}^{\left(2\right)}$ as a function of κ for different *j* values. Inset: $R_{d}^{\left(2\right)}$ versus Hilbert space dimension N for κ=8. Other parameter: α=4π/7. All quantities are dimensionless.

The variation in distance between P(x) and $P_{2}\left(x\right)$, measured by $D_{KL}^{\left(2\right)}$ and $R_{d}^{\left(2\right)}$, with κ for different *j* values, is shown in Figure 8. We see that $D_{KL}^{\left(2\right)}$ and $R_{d}^{\left(2\right)}$ behave in a similar way with increasing κ. For the regular regime with weak kicking strength κ<2, both of them have high values and decrease slowly as κ increases. This means that the coherent states are far from ergodicity in the regular regime. Then, they exhibit a rapid decrease in the region 2≲κ≲5, which corresponds to the crossover from the integrability to full chaos. Finally, for κ>5, as the system becomes globally chaotic, both $D_{KL}^{\left(2\right)}$ and $R_{d}^{\left(2\right)}$ decrease to very small values and are almost independent of κ. Hence, the coherent states are ergodic states in a fully chaotic regime. Moreover, the degree of ergodicity of coherent states in a strongly chaotic regime can be enhanced by increasing the system size, as illustrated in the insets of Figure 8.

Here, an interesting point deserves discussing, namely the connection between the fractal dimensions $D_{q}$ and other quantum chaos probes. Among all detectors of quantum chaos, we focus on spectral form factor (SFF) and out-of-time-ordered correlators (OTOCs). Both of them have been extensively used in numerous recent studies [18,19,20,21,22,100,101,102,103,127,128,129,130,131,132,133,134].

Let us first consider the relation between $D_{q}$ and SFF. The SFF is a powerful tool for detecting the spectral properties of a system and is defined as the Fourier transform of the two-point correlation function of the level density [135]. It is known that the behavior of SFF for integrable systems is drastically different from the chaotic systems, mainly due to the fact that the regular and chaotic systems have different spectral statistics [20]. This means that the SFF can be used as an efficient and sensitive indicator of quantum chaos. As SFF measures the correlation between energy levels, while $D_{q}$ characterizes the complexity of quantum states in a given basis, there is no obvious relation between them. Although, for some particular cases, $D_{q}$ and SFF have been connected in several works [64,136], a more general connection between them is still an open question, beyond the scope of the present work. We will explore this subject in our future work.

We now discuss the comparison of $D_{q}$ with OTOCs. As the main criterion employed to decide whether a quantum system is chaotic or not, OTOC quantifies the sensitivity with respect to the initial condition and information scrambling in quantum systems. It has been demostrated that both the early and late behaviors of OTOC serve as useful diagnostics of quantum chaos [100,101,102,103,137,138]. Since the chaotic dynamics leads to rapid growth and large long-time saturation value in the behavior of OTOC, one can, therefore, expect that the growth rate of the OTOC as well as its long-time saturation value may be correlated with $D_{q}$. However, a more detailed and general connection between them remains an open question. To date, only a formal relationship between $D_{2}$ and OTOCs has been established [139,140].

We finally point out that the degree of extension of a quantum state usually increases with the degree of chaoticity of the system. Hence, we believe that qualitatively similar results should be obtained for generic quantum states and for other quantum systems. Moreover, our main conclusions still hold if the coherent states are expanded to another more localized basis, even if the fractal dimensions are dependent on the choice of the basis.

## 4. Conclusions

In this work, we explored the quantum characters of chaos in the quantum kicked top model by means of multifractal analysis. The kicked top model is a prototype model in the studies of quantum chaos, and its experimental realization has been achieved in several experiments [79,80,81,82]. The signatures of classical chaos have been revealed in various works. It was known that the phase space of classical kicked top has complex structures during the transition from regular to chaotic dynamics. Therefore, understanding how to capture the local chaotic features in quantum system becomes a crucial point in order to understand the quantum-classical correspondence. Although the indicators of quantum chaos, such as level spacing statistics and mean ratio of level spacings, are able to unveil the global signatures of chaos in quantum systems, they cannot detect the local chaotic behaviors. In the present work, with the help of the generalized coherent spin states, we investigated the local chaotic properties of quantum kicked top through the multifractal dimensions of coherent states.

The multifractal analysis of the coherent states is performed by expanding them in orthonormal basis composed by the eigenstates of the Floquet operator. We explicitly demonstrated that the regular regions in the mixed phase space clearly correspond to small values of multifractal dimensions. For the strong chaotic case, the multifractal dimensions exhibit uniform distribution in phase space. Moreover, we have shown that the phase-space-averaged multifractal dimensions serve as indicators of quantum chaos. With kicking strength increasing, the averaged multifractal dimensions undergo a rapid growth, indicating the transition from regular to chaotic dynamics of the system. Coherent states within the strongly chaotic regime become ergodic, with multifractal dimensions tending to unity in the thermodynamic limit, in accordance with the predictions of RMT. However, coherent states’ multifractal dimensions in the regular regime are not equal to zero. Instead, they approach a finite value as the system size goes to infinity.

To get more insight into the multifractal characters of the coherent states and their connections with the underlying chaotic dynamics, we further investigated the probability distribution of the expansion coefficients. Such distribution is expected to follow the so-called $\chi_{\nu }^{2}$ distribution for the fully chaotic systems. We have shown that the deviation between the distribution of coefficients and $\chi_{2}^{2}$ distribution decreases as the kicking strength is increased. For the kicking strengths that lead to the fully chaotic dynamics, the distribution of coefficients exhibits a quite good agreement with $\chi_{2}^{2}$ distribution, implying the strong ergodicity of coherent states. On the contrary, the distribution of coefficients in the regular regime displays a remarkable difference from $\chi_{2}^{2}$ distribution and its long flat tail reveals the localization character of coherent states. In particular, the non-zero fractal dimensions for the regular case can be understood as a consequence of the sharp peak appearing in the probability distribution of coefficients. As the existence of the peak in the distribution of coefficients for the regular regime is a basis-dependent phenomenon, one can therefore expect that the fractal dimensions in regular systems should be equal to zero if a suitable computation basis has been selected. Understanding how to identify an appropriate basis used in the multifractality analysis is an interesting topic for future studies. We also discuss how to measure the distance between the observed distribution of coefficients and the expected $\chi_{2}^{2}$ distribution. We have shown that the transition from regular to chaotic dynamics of the system can be identified by the dramatic decrease in the behavior of different distance measures.

As a final remark, we would like to point out that the recent experimental advances enable a direct observation of multifractality of wave packets in several quantum systems [141,142,143,144]. Hence, the multifractal properties of our studied Floquet system are readily accessible for state-of-the-art experimental platforms.

## Data Availability

Not applicable.
